# Machine Learning-Enhanced MEC Sensors with Feature Engineering for Quantitative Analysis of Multi-Component Toxicants

**DOI:** 10.3390/bios16030144

**Published:** 2026-03-02

**Authors:** Jiaguo Yan, Renxin Liang, Wenqing Yan, Xin Wang

**Affiliations:** 1 PT COSL INDO, China Oilfield Services Limited (COSL), Jakarta 12930, Indonesia; yanjiaguo@cosl.mx; 2 Division of Oilfield Chemicals, China Oilfield Services Limited (COSL), Tianjin 300459, China; liangrenxin@cosl.mx; 3 MOE Key Laboratory of Pollution Processes and Environmental Criteria, Tianjin Key Laboratory of Environmental Remediation and Pollution Control, College of Environmental Science and Engineering, Nankai University, No. 38 Tongyan Road, Jinnan, Tianjin 300350, China; yanwenqing_env@mail.nankai.edu.cn

**Keywords:** machine learning, microbial electrochemical system, mixed toxicants, feature engineering

## Abstract

Accelerated industrialization has caused complex mixed toxicant pollution, where synergistic or antagonistic interactions render conventional detection methods inadequate. Herein, we develop an integrated framework by pioneering the integration of microbial electrochemical systems (MECs) with machine learning (ML) for quantifying formaldehyde, tetracycline, Ag^+^, and Cu^2+^ in multi-component, multi-ratio, and multi-concentration mixtures. MECs generated dynamic current–time (I–t) signals responsive to toxicant stress, though signal overlap from mixed toxicants hindered direct quantification. Guided by toxicokinetics and electrochemical mechanisms, we developed a novel mechanism-driven feature engineering strategy with exclusively original indicators, which extracted 22 multidimensional features capturing instantaneous characteristics, kinetic patterns, and microbial stress-adaptive responses to resolve signal ambiguity, and provided biologically meaningful, high-information feature inputs that effectively bridge electrochemical response signals and ML modeling. Comparative analysis of four ML models (SVM, KNN, PLS, and RF) showed RF outperformed others, achieving R^2^ > 0.9 for all toxicants (formaldehyde: 0.959; tetracycline: 0.934; Ag^+^: 0.936; Cu^2+^: 0.957) with minimized MAE and RMSE. Microbial community analysis identified *Geobacter anodireducens* (71.5%, electroactive for heavy metals) and *Comamonas testosteroni* (12.9%, organic degrader) as key functional taxa, supported by KEGG enzyme abundance data. This work overcomes traditional MEC limitations via innovative feature engineering and pioneering ML integration, providing a rapid, low-cost, and high-accuracy tool for environmental mixed toxicant monitoring.

## 1. Introduction

Accelerated industrialization and the widespread use of chemicals have resulted in pollutants in environmental matrices exhibiting complex characteristics, including multi-component composition, chemical heterogeneity, and trace-level concentrations [1,2]. Emissions from industrial activities, agricultural non-point sources, and domestic waste have led to the accumulation and interaction of diverse toxicants (e.g., heavy metals, organic pollutants, and antibiotics), giving rise to mixed pollution systems. Unlike single pollutants, mixed toxicants may exhibit synergistic or antagonistic interactions in addition to additive effects, substantially complicating environmental risk assessment and posing greater threats to ecosystem stability and human health [3,4]. Consequently, accurate identification and quantification of individual components in mixed pollution systems remain a critical technical bottleneck in environmental monitoring and pollution control.

Currently, the detection methods for mixed toxicants mainly include traditional instrumental analysis [5], bioassays [6] and chemical analysis methods [7]. Microbial electrochemical systems have emerged as promising tools for environmental monitoring due to their low cost, high sensitivity, and environmental compatibility [8,9]. Previous studies have demonstrated that the current–time (I–t) response of microbial electrolysis cells (MECs) is strongly correlated with pollutant concentration, as toxicants inhibit microbial metabolic activity and electron transfer processes [10]. However, interactions among mixed toxicants lead to complex and overlapping I–t response patterns, and traditional single-parameter indicators (e.g., ΔI/I_0_) are insufficient to extract component-specific information, limiting multi-component discrimination.

Machine learning (ML) techniques have demonstrated strong capabilities in high-dimensional data analysis and nonlinear relationship modeling, achieving notable success in pattern recognition and concentration prediction [11,12]. ML can extract informative features from complex nonlinear datasets and reveal latent patterns, offering advantages in resolving signal overlap and interaction effects in mixed systems. Building on these advances, this study integrates MEC sensing with ML to quantify representative environmental toxicants, including formaldehyde, tetracycline, Ag^+^, and Cu^2+^, by constructing multi-component, multi-ratio, and multi-concentration mixed systems and acquiring corresponding I–t response signals. Feature engineering is performed, guided by toxicokinetic considerations, to extract multidimensional indicators, including ΔI/I_0_; optimized Random Forest and other ML models are employed to establish nonlinear mappings between extracted features and individual toxicant concentrations, enabling accurate component identification and quantification in mixed systems. This study aims to overcome the limitations of conventional detection approaches for mixed pollution and to provide a robust framework for rapid and accurate monitoring of environmental mixed toxicants.

## 2. Materials and Methods

### 2.1. Types of Mixed Toxicants

Based on the representativeness of toxicants and the scientific nature of experiments, formaldehyde [13], tetracycline [14], silver nitrate [15] and copper sulfate [15] were selected as typical target toxicants to conduct related toxicity experiments. The maximum concentrations set for the four toxicants are as follows: formaldehyde, 200 ppm; tetracycline, 5 ppm; silver nitrate, 100 ppm; and copper sulfate, 100 ppm. The selection of toxicant concentrations is based on references or preliminary experiments to ensure that MEC-type toxicity sensors respond similarly to the maximum concentrations of the toxicants.

The experimental design includes two treatment modes, individual exposure and mixed exposure, in order to comprehensively investigate the toxic effects of single and combined toxicants. The mixed-exposure system is divided into the following three categories based on combination dimensions, with the specific design as follows:

Binary mixing: A total of 6 toxicant combinations were involved, with each combination set in three volume ratio gradients of 1:1, 2:1 and 1:2, totaling 18 experiments.

Ternary mixtures: A total of 4 types of toxicant combinations are included, and each combination is set with four volumetric ratios of 1:1:1, 2:1:1, 1:2:1 and 1:1:2, resulting in a total of 16 experimental groups.

Quaternary mixture: Volume ratios of 1:1:1:1, 2:1:1:1, 1:2:1:1, 1:1:2:1 and 1:1:1:2 were used for five gradient proportions, totaling 5 experimental groups.

All individual-exposure groups (4 groups) and mixed-exposure groups (39 groups), totaling 43 experimental setups, used 10 concentration equivalents as the maximum concentration of each setup. After gradient dilution, five gradient treatment groups were obtained with relative concentrations of 1, 3, 5, 7 and 10 (the maximum concentration). To ensure the reliability and reproducibility of the experimental results, all treatment groups were set up with parallel experiments.

The specific combination methods of all toxicants, corresponding concentration parameters and detailed gradient dilution schemes have been compiled into a Supplementary Table; see the supporting information (Table S1).

### 2.2. Method of Machine Learning-Enabled MEC Sensors

**Figure 1:**
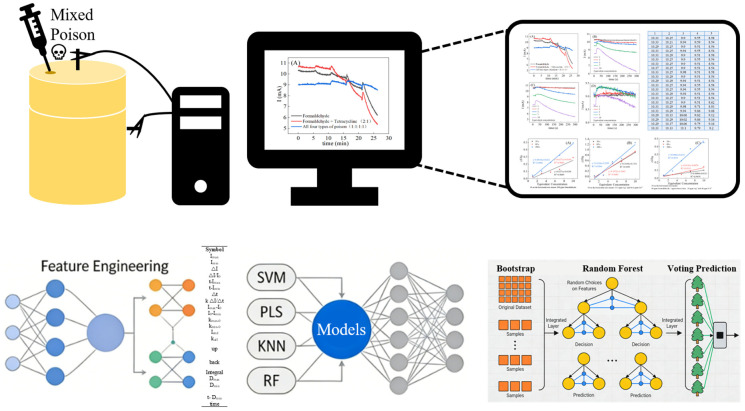
Method of the ML-enabled MEC sensor.

To quantitatively evaluate the toxicological response of MEC sensors and achieve accurate prediction of toxicity levels, this study established an integrated technical framework coupling MEC electrochemical sensing with ML algorithms (Figure 1). The core workflow of this framework was designed as follows: first, target toxicants were injected into the self-fabricated MEC sensor reactor, where the electroactive biofilm immobilized on the working electrode responded to toxicant stress and induced dynamic fluctuations in the sensor’s output current; second, a computer was used to continuously record the real-time current signal of the MEC sensor at fixed time intervals, forming the original time-series dataset for subsequent analysis; third, feature engineering was conducted on the acquired current data to extract key characteristic indicators that could effectively characterize the biofilm’s toxic response; finally, four classical ML algorithms (Support Vector Machine, K-Nearest Neighbors, Partial Least Squares Regression, and Random Forest) were selected to construct toxicity predictive models, and a systematic comparative analysis of the models’ performance was conducted to identify the optimal prediction scheme. The detailed fabrication process of the MEC sensors and the parameter configuration and validation methods of the ML models are elaborated in the following sections.

#### 2.2.1. The Fabrication of MEC Sensors

The MEC sensor comprised a dual-electrode configuration, with a cylindrical carbon brush serving as the working electrode on which the electroactive biofilm was immobilized (Figure S1). The graphite rod measured 6 cm in length and 1 cm in diameter. The counter electrode was constructed from 304N stainless steel mesh (60 mesh) formed into a hollow cylinder, with a diameter of 4.5 cm and a height of 7 cm—this design facilitated the fixation of the graphite rod at the center of the stainless steel mesh cylinder. The reactor was fabricated from a resin bottle with an effective volume of roughly 120 mL, featuring an inner diameter of 4.5 cm and a height of 8 cm. A small aperture was drilled at the top of the container for toxicant injection, which was sealed with a rubber stopper.

The MEC sensors were inoculated using the mixed effluent from acetate-fed microbial fuel cells (MFCs) in our laboratory. A direct-current voltage of 0.7 V was imposed on the MEC sensors, and current data were recorded at a constant interval of 12 s by a self-made data acquisition system [16]. The culture medium was prepared as a 100 mL mixture containing 1 g/L sodium acetate dissolved in 50 mM phosphate buffer solution (PBS: 4.576 g/L Na_2_HPO_4_, 2.132 g/L NaH_2_PO_4_, 0.31 g/L NH_4_Cl, 0.13 g/L KCl, pH = 7.2–7.4), supplemented with 10 mL/L mineral solution (Table S2) and 15 mL/L vitamin solution (Table S3); the formulations of both solutions were detailed in our previous study [17]. A 20 mL aliquot of the culture medium was retained for toxicity testing.

#### 2.2.2. Machine Learning Methods

To achieve accurate modeling and prediction of toxicity-related indicators, this study selected four classical and widely used machine learning algorithms, Support Vector Machine (SVM) [18], K-Nearest Neighbors (KNN) [19], Partial Least Squares Regression (PLS) [20] and Random Forest (RF) [21], to construct predictive models and perform comparative performance analysis. The core principles and technical characteristics of each model are briefly described below.

To ensure the stability, reproducibility and predictive performance of the models, all models were trained and validated using ten-fold cross-validation (10-CV), with the random seed fixed at 42 to ensure consistency in the random processes. The key hyperparameter configurations for each model are as follows:

- SVM: The kernel function uses the Gaussian radial basis function, and the rest of the hyperparameters are set to their default configurations;
- KNN: Set the number of neighbors (K value) to 200, use Euclidean distance as the distance metric and keep the other hyperparameters at their default values;
- PLS: Set the number of extracted principal components to 2, with the remaining hyperparameters following the algorithm’s default settings;
- RF: Set the number of decision trees to 100, while using the algorithm’s default settings for the other hyperparameters.

The complete implementation code for all models, parameter configuration files and detailed operating procedures have all been compiled and archived. For related details, please refer to the supporting information.

### 2.3. Model Evaluation Metrics

To comprehensively and multi-dimensionally evaluate the predictive performance of the model for target concentrations, this study selected mean absolute error (MAE) and root mean square error (RMSE) as the core evaluation metrics. These two indicators quantify the deviation between predicted values and true values from different perspectives: MAE focuses on the overall average deviation level of predictions, while RMSE is more sensitive to extreme prediction errors. Their combination enables a comprehensive assessment of the prediction accuracy and stability of the model, laying a foundation for the in-depth analysis of model performance in subsequent sections.

MAE is defined as the arithmetic mean of the absolute differences between predicted and true values, with the following mathematical expression:

$$MAE=\frac{1}{n}\sum_{i=1}^{n}\left|{\hat{y}}_{i}-y_{i}\right|$$

Here, *n* is the number of samples, *ŷ_i_* is the predicted concentration of the i-th sample and *y_i_* is the corresponding true concentration. MAE is insensitive to outliers, has straightforward calculation logic, and directly reflects the average deviation of model predictions. Smaller MAE values indicate lower average deviations between predicted and true concentrations, suitable for evaluating routine predictive stability. In this study, MAE effectively represented predictive accuracy across concentration gradients, minimizing the influence of extreme deviations from high-concentration samples [22].

RMSE is the square root of the mean square error (MSE), and its mathematical expression is

$$RMSE=\sqrt{\frac{1}{n}\sum_{i=1}^{n}{\left({\hat{y}}_{i}-y_{i}\right)}^{2}}$$

Compared to MAE, RMSE assigns higher weight to larger prediction errors, thereby better reflecting extreme deviations. The magnitude of RMSE not only reflects the average deviation but also highlights severe deviations in individual samples. RMSE shares the same units as concentrations, facilitating intuitive interpretation of prediction errors. In predicting mixed toxicant concentrations, RMSE can specifically evaluate the model’s reliability in predicting high-concentration, strongly synergistic systems. Low RMSE indicates accurate predictions overall and reliable performance in extreme cases, without significant systematic bias [22].

### 2.4. Microbial Community Analysis

Total microbial genomic DNA was extracted from anode biofilm of MEC sensors using the E.Z.N.A.^®^ soil DNA Kit (Omega Bio-tek, Norcross, GA, USA) according to the manufacturer’s instructions. The quality and concentration of DNA were determined by 1.0% agarose gel electrophoresis and a NanoDrop2000 spectrophotometer (Thermo Scientific, Waltham, MA, USA) and kept at −80 °C prior to further use. The hypervariable region V3–V4 of the bacterial 16S rRNA gene was amplified with primer pairs 515F (5′-GTGCCAGCMGCCGCGG-3′) and 806R (5′-GGACTACHVGGGTWTCTAAT-3′) [23] using a T100 Thermal Cycler PCR thermocycler (BIO-RAD, Hercules, CA, USA). The PCR reaction mixture including 5 × 4 μL of Fast Pfu buffer, 2 μL of 2.5 mM dNTPs, 0.8 μL of each primer (5 μM), 0.4 μL of Fast Pfu polymerase, 10 ng of template DNA, and ddH_2_O to a final volume of 20 µL. PCR amplification cycling conditions were as follows: initial denaturation at 95 °C for 3 min, followed by 27 cycles of denaturing at 95 °C for 30 s, annealing at 55 °C for 30 s and extension at 72 °C for 45 s, followed by single extension at 72 °C for 10 min and ending at 4 °C. The PCR product was extracted from 2% agarose gel and purified using the PCR Clean-Up Kit (YuHua, Shanghai, China) according to the manufacturer’s instructions and quantified using Qubit 4.0 (Thermo Fisher Scientific, Waltham, MA, USA).

The illumina sequencing raw dataset was first quality-filtered on the raw data using Trimmomatic, then the primer sequences were identified and removed using Cutadapt, and then the bipartite reads were spliced and chimeras were removed using USEARCH, ultimately obtaining high-quality sequences for subsequent analysis. OTUs were constructed using an identity threshold of 97% and a threshold of 0.005% to analyze the species composition, and then the OTU sequences were annotated.

The relative abundance of a particular strain was calculated from the classification results, which are compared to determine which microorganism had a higher relative abundance. The OTU network analysis was conducted with the Majorbio cloud platform.

## 3. Results and Discussion

### 3.1. Toxic Response

Time–current (I–t) curves showed that, similar to single toxicants, the addition of mixed toxicants to MEC reactors induced current variations, with larger changes observed at higher toxicant concentrations [24]. To quantitatively characterize the nonlinear interactions between mixed toxicants and substantiate the synergistic/antagonistic effects, we calculated the Interaction Index (II) for all mixture systems in this study. The II is defined by the following equation:

$$II=\frac{\Delta I∕I_{o\left(measure\right)}}{\sum {\left(\Delta I∕I_{0}\right)}_{\left(single\right)}}$$

**Figure 2:**
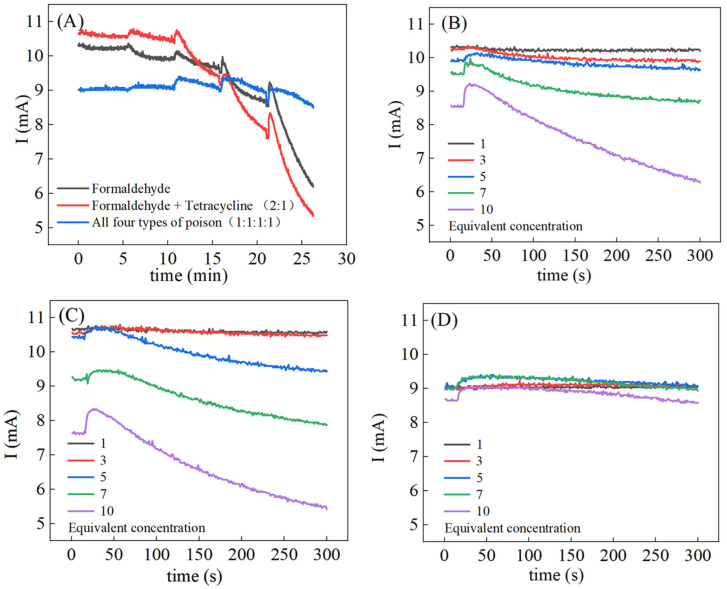
Time–current curves of toxicity experiments: (**A**) Toxicity experiments with a single toxin or a mixture of toxins were conducted five times consecutively, with relative toxin concentrations of 1, 3, 5, 7, and 10, and an experimental duration of 5 min; (**B**) formaldehyde as the toxicant; (**C**) formaldehyde and tetracycline 2:1 as toxicants; (**D**) formaldehyde, tetracycline, Ag^+^ and Cu^2+^ as four toxicants 1:1:1:1 mixed. For the concentration of each specific toxin, refer to Table S1.

Different combinations of toxicants may cause synergistic or antagonistic effects [3] (Figure 2A). Notably, the formaldehyde–tetracycline binary mixture at a volume ratio of 2:1 and an equivalent concentration (EC) of 10 achieved an II value of 1.6, showing a strong synergistic toxic effect, which may be attributed to the oxidation of aldehyde groups by silver ions, which further aggravates the disturbance to the microbial metabolic system. In contrast, the quaternary mixture at an equal volume ratio of 1:1:1:1 and an EC of 10 exhibited an II value as low as 0.18, presenting a significant strong antagonistic effect. At an equivalent concentration of 7, formaldehyde alone produced a ΔI/I_0_ of 0.142, the formaldehyde–tetracycline mixture produced 0.176 (synergistic effect), and the quaternary mixture yielded 0.052 (antagonistic effect). In toxicity experiments, certain toxicant concentrations induced a transient current increase. This transient rise is attributed to microbial stress responses, leading to temporarily enhanced metabolic activity and increased current [25]. At an equivalent concentration of 7 in Figure 2B, the current increase amplitude I_max_ − I_0_/I_0_ is 0.04 and the toxicity response index ΔI/I_0_ is 0.142; at an equivalent concentration of 10 in Figure 2C, the current increase is 0.09 and the toxicity response is 0.39; at an equivalent concentration of 5 in Figure 2D, the current increase is 0.04 and the toxicity response is 0.04. These observations indicate that the transient current increase is not directly proportional to the overall toxicity response, suggesting the existence of concentrations that stimulate microbial metabolism without causing cell death.

**Figure 3:**
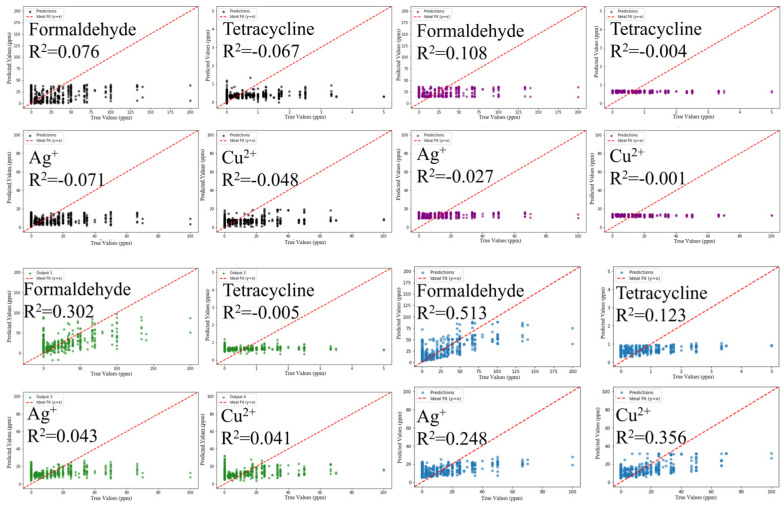
The four models SVM (black dots), KNN (purple dots), PLS (green dots) and RF (blue dots) use time–current curves as input to predict the concentrations of four toxicants. X-axis: true values; Y-axis: predicted values.

Time–current curves were used as input data to train four models SVM, KNN, PLS, and RF to predict concentrations of the four toxicants (Figure 3). Among the 16 fitted curves, only three had an R^2^ greater than 0.3, indicating that the results of direct prediction were not particularly satisfactory. However, among the four models, RF showed relatively superior performance, with an R^2^ of 0.513 for formaldehyde prediction, demonstrating a certain level of interpretability.

**Figure 4:**
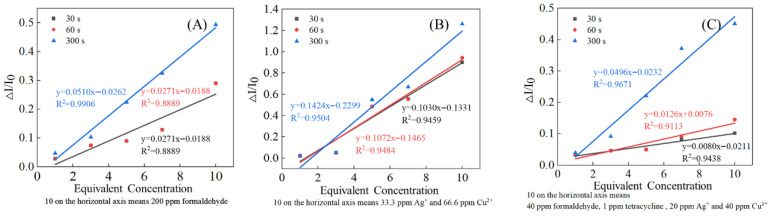
Fitted lines of toxicity response versus concentration for different toxicants. (**A**) Formaldehyde, (**B**) Ag^+^ and Cu^2+^, and (**C**) all 4 poisons.

Data recorded at 30, 60, and 300 s were linearly fitted against equivalent concentrations, showing a consistent linear relationship (Figure 4), as reported previously [10]. From these data, we can see that the curve fitted using the 300 s data has a steeper slope, and the choice of parameters such as ΔI/I_0_ provides ideas for subsequent feature engineering.

### 3.2. Feature Engineering

To overcome limitations of direct modeling of time–current curves—such as interference from redundant information and poor predictive performance—feature engineering was applied as an intermediate step linking electrochemical responses to quantitative concentration predictions [26]. Guided by microbial toxicokinetics [27] and microbial electrochemical mechanisms [28], the dynamic I–t curves were converted into 22 quantifiable features with defined physical and biological significance (Table 1). These features captured instantaneous characteristics, steady-state behavior, and kinetic patterns of the electrochemical response, while encoding relationships among mixed toxicant concentrations, modes of action, and microbial metabolic inhibition, providing a basis for machine learning-based prediction of mixed toxicant concentrations.

The relative current change rate (ΔI/I_0_) is defined as the ratio of the total current change (ΔI = I_max_ − I_min_) to the initial stable current (I_0_). It is a key characteristic reflecting the overall inhibition intensity of toxic substances on the electrochemical activity of microorganisms—its magnitude is directly related to the cumulative toxic effects of mixed toxicants [9].

The integrated area of the I–t curve (integral, ∫I(t) dt) encodes the cumulative extracellular electron transfer capacity of microorganisms during toxicant exposure, reflecting the combined effects of toxicant concentration and exposure time—high concentrations of toxicants usually lead to a rapid decrease in the cumulative amount of electron transfer, ultimately resulting in a smaller integrated area. This feature effectively captures the time-dependent processes in toxicokinetics [29].

In addition, the two binary features (up and back) directly encode the stress and adaptive behaviors of microorganisms: the ‘up’ feature characterizes whether there is a current increase in the early stage of the experiment. When assigned a value of 1, it corresponds to the stress response of microorganisms triggered by toxic stimuli. To resist toxic stress, microorganisms temporarily upregulate metabolic activity to maintain extracellular electron transfer efficiency, ultimately manifesting as a transient rise in current. This phenomenon is more commonly observed in systems with high concentrations of organic toxicants or low doses of mixed heavy metals [25].

The back feature represents whether the current rises again after entering the declining stage. It essentially reflects the metabolic adaptation of microorganisms to low-intensity toxicity: when the toxicant concentration is low or the synergistic inhibitory effect is weak, microorganisms gradually adapt to the toxic environment through their own metabolic regulation, the impaired electron transfer function is gradually restored and the current shows a recovery trend [30].

Collectively, these features encompass multiple response dimensions—including overall inhibitory intensity, long-term trends, cumulative effects, instantaneous dynamics and biological stress behaviors—ensuring comprehensive capture of the intrinsic relationship between mixed toxicant concentrations and microbial electrochemical responses. This provides rich, mechanism-driven informational support for subsequent machine learning modeling.

### 3.3. Machine Learning

**Figure 5:**
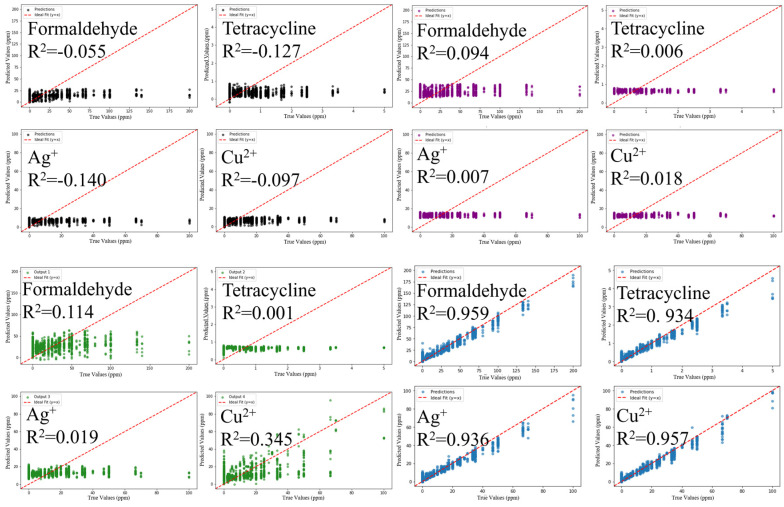
The four models SVM (black dots), KNN (purple dots), PLS (green dots) and RF (blue dots) use feature indicators as input to predict the concentrations of four toxicants. X-axis: true values; Y-axis: predicted values.

Four machine learning algorithms—SVM, KNN, PLS, and RF—were applied to predict concentrations of the four target toxicants using 1290 samples with 22 feature indicators derived from feature engineering (Figure 5). Predictions from the RF model showed effective discrimination of different toxicant components in multi-toxicant systems. Model performance was evaluated by comparing predicted and true concentrations. The coefficients of determination (R^2^) for the RF model were: formaldehyde, 0.959; tetracycline, 0.934; Ag^+^, 0.936; and Cu^2+^, 0.957. The RF model exhibited higher predictive performance than the other three algorithms, achieving R^2^ values above 0.9 for all four toxicants. Among the other three models, only PLS yielded R^2^ values greater than 0.1 for formaldehyde and Cu^2+^.

Model performance was further evaluated using MAE and RMSE (Table 2). The results confirmed that the RF model exhibited the highest predictive performance for all four toxicants. For MAE, prediction errors of the RF model—formaldehyde (4.7046), tetracycline (0.1456), Ag^+^ (2.9381), and Cu^2+^ (2.3252)—were approximately 1/5–1/4 of those of other models, indicating lower average deviations. Regarding RMSE, the RF model showed minimal prediction fluctuations (formaldehyde: 6.7699; tetracycline: 0.2145; Ag^+^: 4.2102; Cu^2+^: 3.4377), indicating improved stability compared with other models (all RMSE > 13). Overall, the RF model, utilizing ensemble learning to capture nonlinear feature–concentration relationships, outperformed SVM, KNN, and PLS in both MAE and RMSE, providing reliable support for precise detection of mixed toxicants.

**Figure 6:**
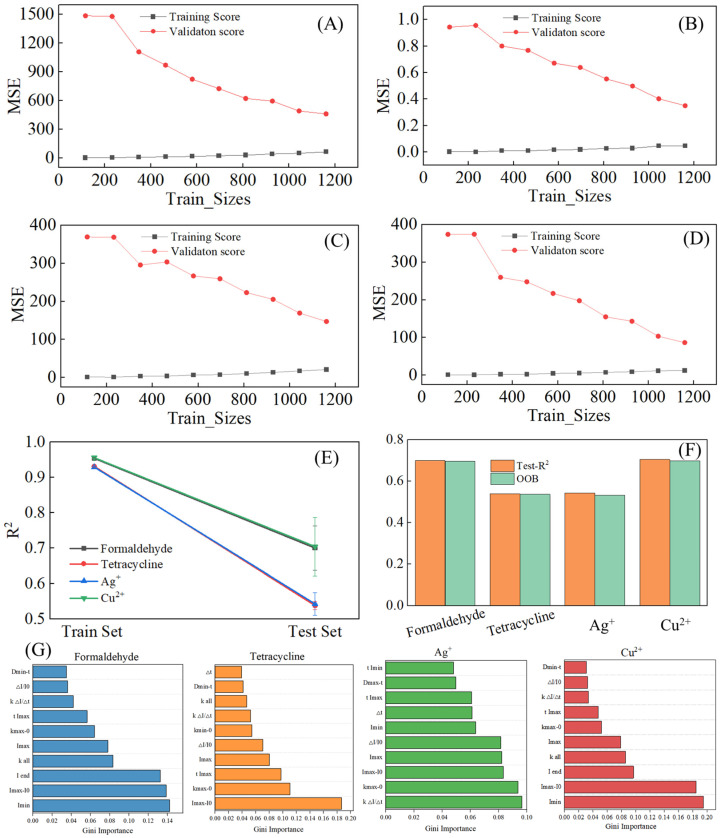
Overfitting validation results of the RF model. (**A**–**D**) Learning curves of the RF model for concentration prediction of formaldehyde, tetracycline, Ag^+^ and Cu^2+^, respectively; (**E**) coefficient of R^2^ distribution of the RF model on the training and test sets; (**F**) comparison between the coefficient of R^2^ of the test set and OOB score of the RF model; (**G**) feature Gini importance of the four toxicants.

Although the RF model has a certain ability to resist overfitting [21], an accuracy greater than 0.9 still prompts us to rigorously verify for overfitting and avoid its risks. Learning curves for formaldehyde, tetracycline, Ag^+^, and Cu^2+^ predictions (Figure 6A–D) showed convergence of training and validation errors as sample size increased. This convergence indicates stable learning of feature–concentration relationships without overfitting, characterized by low training errors coupled with high validation errors [31]. R^2^ distributions for the four toxicants in training and test sets (Figure 6E) showed training R^2^ values above 0.9, indicating strong model fit. Test set R^2^ values were above 0.7 for formaldehyde and Cu^2+^, and above 0.5 for tetracycline and Ag^+^. Small differences from training R^2^ confirmed absence of overfitting and maintenance of predictive ability on independent samples. Test set R^2^ and out-of-bag (OOB) scores (Figure 6F) were compared. OOB scores, computed using samples excluded from the training of individual trees, provide an internal validation metric unique to RF [32]. OOB scores were consistent with test R^2^ values (formaldehyde: 0.005; tetracycline: 0.002; Ag^+^: 0.01; Cu^2+^: 0.006), confirming reliable generalization [32].

To enhance the interpretability of the RF model and clarify its decision-making basis, we calculated the Gini importance of all 22 engineered features (visualized in Figure 6G). Gini importance quantifies a feature’s contribution to reducing node impurity across all decision trees, directly reflecting its role in concentration prediction [33]. Notably, I_max_ − I_0_ (representing the amplitude of current increase in the I–t curve) emerged as a shared high-importance feature for all four toxicants, confirming that the magnitude of current elevation—an intuitive reflection of microbial toxic stress—is a universal and critical predictor for concentration quantification. Meanwhile, the top-ranked features varied among toxicants: for example, ΔI/I_0_ (overall current inhibition intensity) was more important for Cu^2+^ prediction, while kinetic parameters like kΔI/Δt dominated Ag^+^ prediction. This specificity not only validates the model’s ability to capture toxicant-specific response patterns but also provides a basis for distinguishing different toxicants via their characteristic feature contributions.

In summary, convergence of learning curves, consistency between training and test R^2^, and agreement between OOB and test R^2^ confirm the RF model’s stable generalization for mixed toxicant concentration prediction. Additionally, Gini importance analysis further enhances the model’s interpretability: it identifies I_max_ − I_0_ as a universal critical feature for prediction and reveals toxicant-specific feature preferences, verifying that the model relies on meaningful electrochemical response patterns rather than spurious correlations.

### 3.4. Microbial Compositions of MEC Sensors

**Figure 7:**
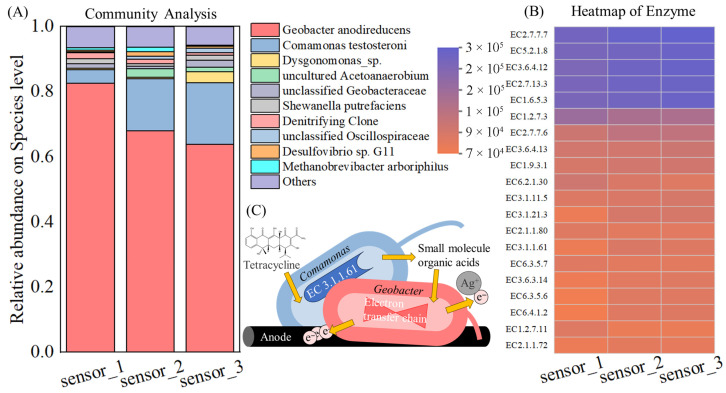
Microbial diversity analysis. (**A**) Microbial community structure at the species level, (**B**) KEGG enzyme abundance heatmap, and (**C**) schematic diagram of interaction mechanism.

From the 16S rRNA gene analysis results (Figure 7), among the top 10 species with the highest relative abundance in the three MEC sensors, *Geobacter anodireducens* exhibited an average relative abundance of up to 71.5%. *Geobacter* is a well-recognized genus of electroactive microorganisms and has been previously applied in Cr^6+^ biosensors [34], which is analogous to the heavy metal toxicity sensing in this study. *Geobacter anodireducens* also possesses core functional traits including dissimilatory metal reduction and extracellular electron transfer [35], enabling its rapid response to heavy metal toxicants. This is also corroborated by the enzyme function heatmap: the high abundance of NADH dehydrogenase (quinone) (EC 1.6.5.3), a key component of the extracellular electron transfer chain in electroactive microorganisms, mediates electron transfer from intracellular NADH to quinone carriers, directly supporting the electrogenesis of *Geobacter* [36]; meanwhile, the expression of glycerol-3-phosphate dehydrogenase (EC 2.7.1.30) and cytochrome c oxidase (EC 1.9.3.1) also confirms the functional link between dissimilatory metal reduction and electron transfer. The former is involved in metabolic regulation under heavy metal stress, while the latter enhances extracellular electron output through the terminal oxidation process of the electron transfer chain, which is highly consistent with the physiological mechanism of *Geobacter* in responding to heavy metal toxicity [37]. Among the top 10 abundant species, unclassified members of *Geobacteraceae* (1.3%, fifth) [38] and *Desulfovibrio* sp. G11 (0.8%, ninth) [39] are also electroactive microorganisms that can directly participate in electrode reactions and thus modulate the current signals.

*Comamonas testosteroni* ranked second with an average relative abundance of 12.9%. This species is not an electroactive microorganism, and there are few reports on its direct interaction with heavy metal ions. Nevertheless, numerous studies have demonstrated that *Comamonas testosteroni* is capable of degrading various organic toxicants such as formaldehyde, PAHs and steroids [40,41,42], making it presumably the key microbial species responsible for the distinct response to organic toxicants in this study. Enzymological evidence supporting this function was also found in the enzyme function heatmap: the high abundance of carboxylesterase (EC 3.1.1.61), pyruvate synthase (EC 1.2.7.11), and acetyl-CoA carboxylase (EC 6.4.1.2). Among these, carboxylesterase can directly catalyze the hydrolytic degradation of organic toxicants (e.g., esters and PAH derivatives), while pyruvate synthase and acetyl-CoA carboxylase provide energy and material basis for organic toxicant degradation by regulating glycolysis and fatty acid synthesis pathways, further confirming the core role of *Comamonas testosteroni* in response to organic toxicants [43]. Although the metabolic activities of *Comamonas testosteroni* do not directly induce alterations in electrical current signals, this species shares the same ecological niche with electroactive microorganisms, leading to potential metabolic interplays between them. Enzymological characteristics implying metabolic interplay between species can also be observed in the enzyme heatmap: the co-high expression of *Geobacter*-associated electron transfer enzymes (EC 1.6.5.3 and EC 1.9.3.1) and *Comamonas*-associated organic degradation enzymes (EC 3.1.1.61 and EC 6.4.1.2) in samples indicates that the two microbial groups may interact through the sharing of metabolic intermediates (e.g., pyruvate and acetyl-CoA). Specifically, small molecules produced by *Comamonas* during organic toxicant degradation can serve as substrates for electron transfer in *Geobacter*, while the electrogenesis of *Geobacter* maintains the redox homeostasis of the microenvironment, which in turn promotes the metabolic activity of *Comamonas*. In addition, the coordinated expression of asparagine synthase (EC 6.3.5.6) and glutamine synthetase (EC 6.3.5.7) reflects the complementarity of nitrogen metabolism pathways, further supporting the hypothesis of metabolic interplay under niche overlap [44]. These interplays ultimately result in variations in current signals, and the effects induced by different combinations of toxicant concentrations vary considerably. Such variations are reflected in the differences in response time, stress response and integrated area of the current signals, which are further used as the core indicators of feature engineering for the performance demonstration of subsequent machine learning models.

## 4. Conclusions

An integrated framework coupling microbial electrolysis cell (MEC) sensors with machine learning was developed to quantify formaldehyde, tetracycline, Ag^+^, and Cu^2+^ in multi-component, multi-ratio, and multi-concentration mixtures. Guided by microbial toxicokinetic principles and electrochemical mechanisms, 22 informative features were extracted from dynamic current–time signals, capturing instantaneous electrochemical characteristics, steady-state behaviors, kinetic patterns, and microbial stress-adaptive responses. Comparative analysis of four classical machine learning models (SVM, KNN, PLS, and RF) demonstrated that the RF model outperformed the others significantly, achieving coefficients of determination (R^2^) > 0.9 for all target toxicants (formaldehyde: 0.959; tetracycline: 0.934; Ag^+^: 0.936; Cu^2+^: 0.957) with markedly reduced mean absolute error (MAE) and root mean square error (RMSE). Rigorous validations including ten-fold cross-validation, learning curve convergence, consistency between training and test set performances, and out-of-bag (OOB) score agreement confirmed the RF model’s robust generalization without overfitting. Microbial community analysis revealed that the MEC anode biofilm was dominated by *Geobacter anodireducens* (71.5%), a key electroactive microbe with dissimilatory metal reduction and extracellular electron transfer capabilities that underpinned heavy metal sensing, and *Comamonas testosteroni* (12.9%), an organic toxicant-degrading bacterium whose metabolic interactions with electroactive microbes contributed to distinct electrochemical signals for organic–inorganic mixed systems. This observation is further supported by the KEGG enzyme abundance results. This work addresses the critical limitation of conventional MEC sensing in resolving complex signals from mixed toxicant systems, providing a rapid, low-cost, and high-accuracy monitoring strategy. Future efforts will focus on expanding the scope of target toxicants, miniaturizing MEC sensor devices for portable on-site detection, and conducting systematic validation in real-world water matrices like surface water or industrial wastewater to address matrix interference, followed by large-scale field validation to enhance the practical applicability of this framework for environmental risk assessment and pollution control.

## Figures and Tables

**Table 1:** Indicators selected by feature engineering.

Symbol	Meaning	Calculation
I_max_	Maximum current during the experiment	——
I_min_	Minimum current during the experiment	——
ΔI	Current change	I_max_ − I_min_
ΔI/I_0_	Relative current change	$\frac{\Delta I}{I_{0}}$, I_0_ is the initial current
t − I_max_	Time of maximum current	——
t − I_min_	Time of minimum current	——
Δt	Time difference of maximum current difference	t − I_max_ – t − I_min_
k ΔI/Δt	Slope of secant of maximum current difference	$\frac{\Delta I}{\Delta t}$
I_max_ − I_0_	Current increment	I_max_ − I_0_
I_0_ − I_min_	Current decrement	I_0_ − I_min_
k_max-0_	Slope of secant from start to maximum current	$\frac{I_{max}-I_{0}}{{t-I}_{max}}$
k_min-0_	Slope of secant from start to minimum current	$\frac{I_{min}-I_{0}}{{t-I}_{min}}$
I_end_	End current	——
time	Duration of experiment	——
k_all_	Slope of secant connecting start and end points	$\frac{I_{end}-I_{0}}{time}$
up	Whether current rises at the start of the experiment	Occurs = 1; does not occur = 0
back	Whether current rises in the declining phase	Occurs = 1; does not occur = 0
Integral	Integral area	$\int_{0}^{time}I\left(t\right)dt$; I(t) is the time–current curve
D_max_	Maximum derivative	${\left(\frac{dI\left(t\right)}{dt}\right)}_{max}$
D_min_	Minimum derivative	${\left(\frac{dI\left(t\right)}{dt}\right)}_{min}$
t − D_max_	Time of maximum derivative	——
t − D_min_	Time of minimum derivative	——

**Table 2:** Comparison of different toxicants’ prediction performance evaluation for various models.

		Formaldehyde	Tetracycline	Ag^+^	Cu^2+^
SVM	MAE	22.8395	0.5952	11.9346	11.7178
	RMSE	34.044	0.8777	17.6507	17.3149
	R^2^	−0.0548	−0.1266	−0.1398	−0.0971
KNN	MAE	23.0699	0.6359	12.6767	12.5076
	RMSE	31.7216	0.8305	16.6009	16.5105
	R^2^	0.0935	0.0059	0.007	0.0177
PLS	MAE	23.0052	0.639	12.4264	10.2744
	RMSE	31.3562	0.8326	16.5019	13.4872
	R^2^	0.1143	0.0009	0.0188	0.3445
RF	MAE	4.7046	0.1456	2.9381	2.3252
	RMSE	6.7699	0.2145	4.2102	3.4377
	R^2^	0.9587	0.9337	0.9361	0.9574

## Data Availability

The original contributions presented in the study are included in the article; further inquiries can be directed to the corresponding author.

## Supplementary Materials

The following supporting information can be downloaded at: https://www.mdpi.com/article/10.3390/bios16030144/s1, Table S1: The correspondence between equivalent concentrations of different combinations and the concentrations of toxins and codes; Table S2: Components of mineral solution; Table S3: Components of vitamin solution; Figure S1: Reactor diagram: (A) overall structure, (B) anode graphite rod, and (C) cathode stainless steel mesh; Code of four models.
