# Supplementary material for: Machine Learning-Enhanced MEC Sensors with Feature Engineering for Quantitative Analysis of Multi-Component Toxicants
Source: Biosensors (Basel). 2026 Mar 2;16(3):144. doi: 10.3390/bios16030144 (PMC13023910; doi:10.3390/bios16030144)
Supplement: Supplementary file 1 [file biosensors-16-00144-s001.zip › biosensors-4166094-supplementary.pdf]

Supplementary Information

Table S1 The correspondence between equivalent concentrations of different combinations and the concentrations of toxins and codes

Concentration (ppm)		Equivalent Concentration			1				3				5				7				10				
Poisons Type				Ratio	Formaldehyde	Tetracycline	Ag^{+}	Cu^{2+}	Formaldehyde	Tetracycline	Ag^{+}	Cu^{2+}	Formaldehyde	Tetracycline	Ag^{+}	Cu^{2+}	Formaldehyde	Tetracycline	Ag^{+}	Cu^{2+}	Formaldehyde	Tetracycline	Ag^{+}	Cu^{2+}	Code
Formaldehyde					20.00	0.00	0.00	0.00	60.00	0.00	0.00	0.00	100.00	0.00	0.00	0.00	140.00	0.00	0.00	0.00	200.00	0.00	0.00	0.00	A
Formaldehyde	Tetracycline			1:1	10.00	0.25	0.00	0.00	30.00	0.75	0.00	0.00	50.00	1.25	0.00	0.00	70.00	1.75	0.00	0.00	100.00	2.50	0.00	0.00	AB1
Formaldehyde	Tetracycline			2:1	13.33	0.17	0.00	0.00	40.00	0.50	0.00	0.00	66.67	0.83	0.00	0.00	93.33	1.17	0.00	0.00	133.33	1.67	0.00	0.00	AB2
Formaldehyde	Tetracycline			1:2	6.67	0.33	0.00	0.00	20.00	1.00	0.00	0.00	33.33	1.67	0.00	0.00	46.67	2.33	0.00	0.00	66.67	3.33	0.00	0.00	AB3
Formaldehyde	Tetracycline	Ag^{+}		1:1:1	6.67	0.17	3.33	0.00	20.00	0.50	10.00	0.00	33.33	0.83	16.67	0.00	46.67	1.17	23.33	0.00	66.67	1.67	33.33	0.00	ABC1
Formaldehyde	Tetracycline	Ag^{+}		2:1:1	10.00	0.13	2.50	0.00	30.00	0.38	7.50	0.00	50.00	0.63	12.50	0.00	70.00	0.88	17.50	0.00	100.00	1.25	25.00	0.00	ABC2
Formaldehyde	Tetracycline	Ag^{+}		1:2:1	5.00	0.25	2.50	0.00	15.00	0.75	7.50	0.00	25.00	1.25	12.50	0.00	35.00	1.75	17.50	0.00	50.00	2.50	25.00	0.00	ABC3
Formaldehyde	Tetracycline	Ag^{+}		1:1:2	5.00	0.13	5.00	0.00	15.00	0.38	15.00	0.00	25.00	0.63	25.00	0.00	35.00	0.88	35.00	0.00	50.00	1.25	50.00	0.00	ABC4
Formaldehyde	Tetracycline	Cu^{2+}		1:1:1	6.67	0.17	0.00	3.33	20.00	0.50	0.00	10.00	33.33	0.83	0.00	16.67	46.67	1.17	0.00	23.33	66.67	1.67	0.00	33.33	ABD1
Formaldehyde	Tetracycline	Cu^{2+}		2:1:1	10.00	0.13	0.00	2.50	30.00	0.38	0.00	7.50	50.00	0.63	0.00	12.50	70.00	0.88	0.00	17.50	100.00	1.25	0.00	25.00	ABD2
Formaldehyde	Tetracycline	Cu^{2+}		1:2:1	5.00	0.25	0.00	2.50	15.00	0.75	0.00	7.50	25.00	1.25	0.00	12.50	35.00	1.75	0.00	17.50	50.00	2.50	0.00	25.00	ABD3
Formaldehyde	Tetracycline	Cu^{2+}		1:1:2	5.00	0.13	0.00	5.00	15.00	0.38	0.00	15.00	25.00	0.63	0.00	25.00	35.00	0.88	0.00	35.00	50.00	1.25	0.00	50.00	ABD4
Formaldehyde	Ag^{+}			1:1	10.00	0.00	5.00	0.00	30.00	0.00	15.00	0.00	50.00	0.00	25.00	0.00	70.00	0.00	35.00	0.00	100.00	0.00	50.00	0.00	AC1
Formaldehyde	Ag^{+}			2:1	13.33	0.00	3.33	0.00	40.00	0.00	10.00	0.00	66.67	0.00	16.67	0.00	93.33	0.00	23.33	0.00	133.33	0.00	33.33	0.00	AC2
Formaldehyde	Ag^{+}			1:2	6.67	0.00	6.67	0.00	20.00	0.00	20.00	0.00	33.33	0.00	33.33	0.00	46.67	0.00	46.67	0.00	66.67	0.00	66.67	0.00	AC3
Formaldehyde	Ag^{+}	Cu^{2+}		1:1:1	6.67	0.00	3.33	3.33	20.00	0.00	10.00	10.00	33.33	0.00	16.67	16.67	46.67	0.00	23.33	23.33	66.67	0.00	33.33	33.33	ACD1
Formaldehyde	Ag^{+}	Cu^{2+}		2:1:1	10.00	0.00	2.50	2.50	30.00	0.00	7.50	7.50	50.00	0.00	12.50	12.50	70.00	0.00	17.50	17.50	100.00	0.00	25.00	25.00	ACD2
Formaldehyde	Ag^{+}	Cu^{2+}		1:2:1	5.00	0.00	5.00	2.50	15.00	0.00	15.00	7.50	25.00	0.00	25.00	12.50	35.00	0.00	35.00	17.50	50.00	0.00	50.00	25.00	ACD3
Formaldehyde	Ag^{+}	Cu^{2+}		1:1:2	5.00	0.00	2.50	5.00	15.00	0.00	7.50	15.00	25.00	0.00	12.50	25.00	35.00	0.00	17.50	35.00	50.00	0.00	25.00	50.00	ACD4
Formaldehyde	Cu^{2+}			1:1	10.00	0.00	0.00	5.00	30.00	0.00	0.00	15.00	50.00	0.00	0.00	25.00	70.00	0.00	0.00	35.00	100.00	0.00	0.00	50.00	AD1
Formaldehyde	Cu^{2+}			2:1	13.33	0.00	0.00	3.33	40.00	0.00	0.00	10.00	66.67	0.00	0.00	16.67	93.33	0.00	0.00	23.33	133.33	0.00	0.00	33.33	AD2
Formaldehyde	Cu^{2+}			1:2	6.67	0.00	0.00	6.67	20.00	0.00	0.00	20.00	33.33	0.00	0.00	33.33	46.67	0.00	0.00	46.67	66.67	0.00	0.00	66.67	AD3
Formaldehyde	Tetracycline	Ag^{+}	Cu^{2+}	1:1:1:1	5.00	0.13	2.50	2.50	15.00	0.38	7.50	7.50	25.00	0.63	12.50	12.50	35.00	0.88	17.50	17.50	50.00	1.25	25.00	25.00	ALL1
Formaldehyde	Tetracycline	Ag^{+}	Cu^{2+}	2:1:1:1	8.00	0.10	2.00	2.00	24.00	0.30	6.00	6.00	40.00	0.50	10.00	10.00	56.00	0.70	14.00	14.00	80.00	1.00	20.00	20.00	ALL2
Formaldehyde	Tetracycline	Ag^{+}	Cu^{2+}	1:2:1:1	4.00	0.20	2.00	2.00	12.00	0.60	6.00	6.00	20.00	1.00	10.00	10.00	28.00	1.40	14.00	14.00	40.00	2.00	20.00	20.00	ALL3
Formaldehyde	Tetracycline	Ag^{+}	Cu^{2+}	1:1:2:1	4.00	0.10	4.00	2.00	12.00	0.30	12.00	6.00	20.00	0.50	20.00	10.00	28.00	0.70	28.00	14.00	40.00	1.00	40.00	20.00	ALL4
Formaldehyde	Tetracycline	Ag^{+}	Cu^{2+}	1:1:1:2	4.00	0.10	2.00	4.00	12.00	0.30	6.00	12.00	20.00	0.50	10.00	20.00	28.00	0.70	14.00	28.00	40.00	1.00	20.00	40.00	ALL5
Tetracycline					0.00	0.50	0.00	0.00	0.00	1.50	0.00	0.00	0.00	2.50	0.00	0.00	0.00	3.50	0.00	0.00	0.00	5.00	0.00	0.00	B
Tetracycline	Ag^{+}			1:1	0.00	0.25	5.00	0.00	0.00	0.75	15.00	0.00	0.00	1.25	25.00	0.00	0.00	1.75	35.00	0.00	0.00	2.50	50.00	0.00	BC1
Tetracycline	Ag^{+}			2:1	0.00	0.33	3.33	0.00	0.00	1.00	10.00	0.00	0.00	1.67	16.67	0.00	0.00	2.33	23.33	0.00	0.00	3.33	33.33	0.00	BC2
Tetracycline	Ag^{+}			1:2	0.00	0.17	6.67	0.00	0.00	0.50	20.00	0.00	0.00	0.83	33.33	0.00	0.00	1.17	46.67	0.00	0.00	1.67	66.67	0.00	BC3
Tetracycline	Ag^{+}	Cu^{2+}		1:1:1	0.00	0.17	3.33	3.33	0.00	0.50	10.00	10.00	0.00	0.83	16.67	16.67	0.00	1.17	23.33	23.33	0.00	1.67	33.33	33.33	BCD1
Tetracycline	Ag^{+}	Cu^{2+}		2:1:1	0.00	0.25	2.50	2.50	0.00	0.75	7.50	7.50	0.00	1.25	12.50	12.50	0.00	1.75	17.50	17.50	0.00	2.50	25.00	25.00	BCD2
Tetracycline	Ag^{+}	Cu^{2+}		1:2:1	0.00	0.13	5.00	2.50	0.00	0.38	15.00	7.50	0.00	0.63	25.00	12.50	0.00	0.88	35.00	17.50	0.00	1.25	50.00	25.00	BCD3
Tetracycline	Ag^{+}	Cu^{2+}		1:1:2	0.00	0.13	2.50	5.00	0.00	0.38	7.50	15.00	0.00	0.63	12.50	25.00	0.00	0.88	17.50	35.00	0.00	1.25	25.00	50.00	BCD4
Tetracycline	Cu^{2+}			1:1	0.00	0.25	0.00	5.00	0.00	0.75	0.00	15.00	0.00	1.25	0.00	25.00	0.00	1.75	0.00	35.00	0.00	2.50	0.00	50.00	BD1
Tetracycline	Cu^{2+}			2:1	0.00	0.33	0.00	3.33	0.00	1.00	0.00	10.00	0.00	1.67	0.00	16.67	0.00	2.33	0.00	23.33	0.00	3.33	0.00	33.33	BD2
Tetracycline	Cu^{2+}			1:2	0.00	0.17	0.00	6.67	0.00	0.50	0.00	20.00	0.00	0.83	0.00	33.33	0.00	1.17	0.00	46.67	0.00	1.67	0.00	66.67	BD3
Ag^{+}					0.00	0.00	10.00	0.00	0.00	0.00	30.00	0.00	0.00	0.00	50.00	0.00	0.00	0.00	70.00	0.00	0.00	0.00	100.00	0.00	C
Ag^{+}	Cu^{2+}			1:1	0.00	0.00	5.00	5.00	0.00	0.00	15.00	15.00	0.00	0.00	25.00	25.00	0.00	0.00	35.00	35.00	0.00	0.00	50.00	50.00	CD1
Ag^{+}	Cu^{2+}			2:1	0.00	0.00	6.67	3.33	0.00	0.00	20.00	10.00	0.00	0.00	33.33	16.67	0.00	0.00	46.67	23.33	0.00	0.00	66.67	33.33	CD2
Ag^{+}	Cu^{2+}			1:2	0.00	0.00	3.33	6.67	0.00	0.00	10.00	20.00	0.00	0.00	16.67	33.33	0.00	0.00	23.33	46.67	0.00	0.00	33.33	66.67	CD3
Cu^{2+}					0.00	0.00	0.00	10.00	0.00	0.00	0.00	30.00	0.00	0.00	0.00	50.00	0.00	0.00	0.00	70.00	0.00	0.00	0.00	100.00	D

Table S2 Components of mineral solution

Component	Content (g/L)	Component	Content (g/L)
Nitrilotriacetic acid	1.5	$MgSO_4$	3
$MnSO_4 \cdot H_2O$	0.5	NaCl	1
$FeSO_4 \cdot 7H_2O$	0.1	$CaCl_2 \cdot 2H_2O$	0.1
$CoCl_2 \cdot 6H_2O$	0.1	$ZnCl_2$	0.13
$CuSO_4 \cdot 5H_2O$	0.01	$AlK(SO_4)_2 \cdot 12H_2O$	0.01
H_3BO_3	0.01	$Na_2MoO_4 \cdot 2H_2O$	0.025
$NiCl_2 \cdot 6H_2O$	0.024	$Na_2WO_4 \cdot 2H_2O$	0.025

Table S3 Components of vitamin solution

Component	Content (g/L)	Component	Content (g/L)
Biotin	0.002	Vitamin B_5	0.005
Vitamin B_{12}	0.0001	p-Aminobenzoic acid	0.005
Lipoic acid	0.005	Vitamin B_3	0.005
Vitamin B_1	0.005	Vitamin B_2	0.005
Vitamin B_6	0.01	Folic acid	0.002

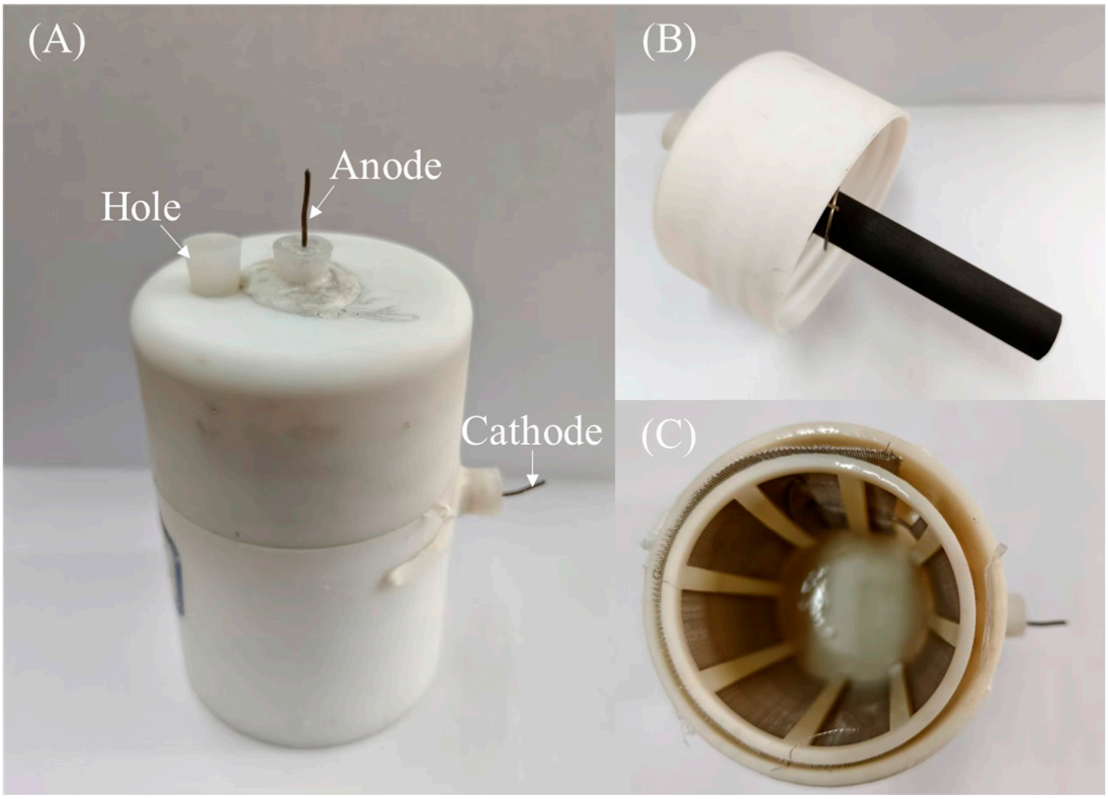

Figure S1. Reactor diagram: (A) overall structure (B) anode graphite rod and (C) cathode stainless steel mesh.

1.1 The model code of SVM

```
import pandas as pd
import numpy as np
import matplotlib.pyplot as plt
from sklearn.model_selection import KFold
from sklearn.svm import SVR
from sklearn.multioutput import MultiOutputRegressor
from sklearn.metrics import r2_score, mean_absolute_error,
mean_squared_error

# Read data
file_path = r"C:\Users\gooddata.xlsx"
df = pd.read_excel(file_path, sheet_name="Sheet1")

# Split features and target variables
X = df.iloc[:, :22]
X.columns = X.columns.astype(str)
y = df.iloc[:, 23:27]

# Configure 10-fold cross-validation
kf = KFold(n_splits=10, shuffle=True, random_state=42)

# Store evaluation metrics for each dimension
r2_scores = [[] for _ in range(y.shape[1])]
mae_scores = [[] for _ in range(y.shape[1])]
rmse_scores = [[] for _ in range(y.shape[1])]
true_vals = [[] for _ in range(y.shape[1])]
pred_vals = [[] for _ in range(y.shape[1])]

# Initialize SVR model (multi-output regression)
svr_base = SVR(kernel='rbf', C=1.0, gamma='scale')
model = MultiOutputRegressor(svr_base)

# Cross-validation training and evaluation
for train_idx, test_idx in kf.split(X):
    X_train, X_test = X.iloc[train_idx], X.iloc[test_idx]
    y_train, y_test = y.iloc[train_idx], y.iloc[test_idx]

    # Train the model
    model.fit(X_train, y_train)
    # Make predictions
    y_pred = model.predict(X_test)

    # Calculate evaluation metrics for each output dimension
    for i in range(y.shape[1]):
        r2 = r2_score(y_test.iloc[:, i], y_pred[:, i])
        mae = mean_absolute_error(y_test.iloc[:, i], y_pred[:, i])
        rmse = np.sqrt(mean_squared_error(y_test.iloc[:, i], y_pred[:, i]))

        r2_scores[i].append(r2)
```

```python
            mae_scores[i].append(mae)
            rmse_scores[i].append(rmse)

            true_vals[i].extend(y_test.iloc[:, i].values)
            pred_vals[i].extend(y_pred[:, i])

# Print cross-validation results
print("\n10-Fold Cross-Validation Results:")
for i in range(y.shape[1]):
    avg_r2 = np.mean(r2_scores[i])
    std_r2 = np.std(r2_scores[i])

    avg_mae = np.mean(mae_scores[i])
    std_mae = np.std(mae_scores[i])

    avg_rmse = np.mean(rmse_scores[i])
    std_rmse = np.std(rmse_scores[i])

    print(f"\nOutput Dimension {i+1} (Column {24+i}):")
    print(f"R²: {avg_r2:.4f} ± {std_r2:.4f}")
    print(f"MAE: {avg_mae:.4f} ± {std_mae:.4f}")
    print(f"RMSE: {avg_rmse:.4f} ± {std_rmse:.4f}")

# Plot true values vs predicted values comparison chart
plt.figure(figsize=(15, 10))
plt.suptitle('True Values vs Predicted Values (10-Fold Average)', fontsize=14,
y=1.02)

for i in range(y.shape[1]):
    plt.subplot(2, 2, i+1)
    true = np.array(true_vals[i])
    pred = np.array(pred_vals[i])

    # Sort to plot trend line
    plot_df = pd.DataFrame({'True': true, 'Pred': pred}).sort_values('True')
    true_sorted = plot_df['True'].values
    pred_sorted = plot_df['Pred'].values

    # Scatter plot
    plt.scatter(true, pred, alpha=0.6, s=30, color='black', label='Predicted
Values')
    # Prediction trend line
    plt.plot(true_sorted, pred_sorted, 'b-', label='Prediction Trend',
linewidth=1)
    # Ideal fit line (y=x)
    val_min = min(true.min(), pred.min())
    val_max = max(true.max(), pred.max())
    buffer = (val_max - val_min) * 0.05
    plt.plot([val_min - buffer, val_max + buffer], [val_min - buffer, val_max
+ buffer],
```

```
                      'r--', linewidth=2, label='Ideal Fit Line')

        # Axis range
        plt.xlim(val_min - buffer, val_max + buffer)
        plt.ylim(val_min - buffer, val_max + buffer)

        plt.xlabel('True Values', fontsize=11)
        plt.ylabel('Predicted Values', fontsize=11)
        plt.title(f'Output Dimension {i+1}', fontsize=12, pad=10)
        plt.legend(fontsize=10)

plt.tight_layout()
plt.show()
```

1.2 The model code of KNN

```
import pandas as pd
import numpy as np
import matplotlib.pyplot as plt
from sklearn.model_selection import KFold, cross_val_predict
from sklearn.neighbors import KNeighborsRegressor
from sklearn.metrics import mean_squared_error, mean_absolute_error, r2_score

# Read data
file_path = r"C:\Users\gooddata.xlsx"
df = pd.read_excel(file_path, sheet_name="Sheet1")

# Split features and target variables
X = df.iloc[:, :22]
X.columns = X.columns.astype(str)
y = df.iloc[:, 23:27]

# Initialize KNN regression model
knn = KNeighborsRegressor(n_neighbors=200)

# 10-fold cross-validation configuration
kf = KFold(n_splits=10, shuffle=True, random_state=60)

# Cross-validation prediction
y_pred = cross_val_predict(knn, X, y, cv=kf)

# Calculate evaluation metrics for each dimension
mse = mean_squared_error(y, y_pred, multioutput='raw_values')
rmse = np.sqrt(mse)
mae = mean_absolute_error(y, y_pred, multioutput='raw_values')
r2 = r2_score(y, y_pred, multioutput='raw_values')

# Print evaluation results
print("Multi-dimensional Evaluation Metrics:")
for i in range(4):
    print(f"\nOutput Dimension {i+1}:")
    print(f'MSE:    {mse[i]:.4f}')
    print(f'RMSE: {rmse[i]:.4f}')
    print(f'MAE:    {mae[i]:.4f}')
    print(f'R²:      {r2[i]:.4f}')

# Visualize true values vs predicted values
plt.figure(figsize=(15, 10))
for i in range(4):
    plt.subplot(2, 2, i+1)

    # Get data for current dimension
    true_vals = y.iloc[:, i]
    pred_vals = y_pred[:, i]

```

```
        # Calculate coordinate range
        min_val = min(true_vals.min(), pred_vals.min())
        max_val = max(true_vals.max(), pred_vals.max())
        buffer = (max_val - min_val) * 0.05

        # Plot scatter plot and reference line
        plt.scatter(true_vals, pred_vals, alpha=0.6)
        plt.plot([min_val - buffer, max_val + buffer],
                 [min_val - buffer, max_val + buffer], 'r--')

        # Set axis limits
        plt.xlim(min_val - buffer, max_val + buffer)
        plt.ylim(min_val - buffer, max_val + buffer)

        plt.xlabel('True Values')
        plt.ylabel('Predicted Values')
        plt.title(f'Output Dimension {i+1}')

plt.tight_layout()
plt.savefig('prediction_visualization.png', dpi=300, bbox_inches='tight')
plt.show()
```

1.3 The model code of PLS

```
import pandas as pd
import numpy as np
import matplotlib.pyplot as plt
from sklearn.cross_decomposition import PLSRegression
from sklearn.model_selection import KFold
from sklearn.metrics import r2_score, mean_squared_error, mean_absolute_error
from sklearn.preprocessing import StandardScaler

# Read data
file_path = r"C:\Users\gooddata.xlsx"
df = pd.read_excel(file_path, sheet_name="Sheet1")

# Split features and target variables
X = df.iloc[:, :22].values
y = df.iloc[:, 23:27].values
n_outputs = y.shape[1]

# Standardize data
scaler_X = StandardScaler()
scaler_y = StandardScaler()
X_scaled = scaler_X.fit_transform(X)
y_scaled = scaler_y.fit_transform(y)

# Initialize 10-fold cross-validation
kf = KFold(n_splits=10, shuffle=True, random_state=8)
pls = PLSRegression(n_components=2)

# Store true and predicted values for each dimension
y_true_list = [[] for _ in range(n_outputs)]
y_pred_list = [[] for _ in range(n_outputs)]

# Cross-validation training and prediction
for train_idx, test_idx in kf.split(X_scaled):
    X_train, X_test = X_scaled[train_idx], X_scaled[test_idx]
    y_train, y_test = y_scaled[train_idx], y_scaled[test_idx]

    # Train the model
    pls.fit(X_train, y_train)

    # Make predictions and inverse transform to original scale
    y_pred_scaled = pls.predict(X_test)
    y_pred = scaler_y.inverse_transform(y_pred_scaled)
    y_true = scaler_y.inverse_transform(y_test)

    # Store data
    for i in range(n_outputs):
        y_true_list[i].extend(y_true[:, i])
        y_pred_list[i].extend(y_pred[:, i])

```

```
# Calculate evaluation metrics
metrics = {}
for i in range(n_outputs):
    y_true_arr = np.array(y_true_list[i])
    y_pred_arr = np.array(y_pred_list[i])

    r2 = r2_score(y_true_arr, y_pred_arr)
    rmse = np.sqrt(mean_squared_error(y_true_arr, y_pred_arr))
    mae = mean_absolute_error(y_true_arr, y_pred_arr)

    metrics[f"Dimension {i+1}"] = {"R²": r2, "RMSE": rmse, "MAE": mae}

# Print results
print("Regression Evaluation Metrics:")
for dim, val in metrics.items():
    print(f"{dim}:")
    print(f"   R²: {val['R²']:.4f}")
    print(f"   RMSE: {val['RMSE']:.4f}")
    print(f"   MAE: {val['MAE']:.4f}\n")

# Visualize true values vs predicted values
plt.figure(figsize=(15, 10))
plt.suptitle('True Values vs Predicted Values', fontsize=14, y=1.02)

for i in range(n_outputs):
    plt.subplot(2, 2, i+1)

    y_true_arr = np.array(y_true_list[i])
    y_pred_arr = np.array(y_pred_list[i])

    # Calculate axis range
    min_val = min(y_true_arr.min(), y_pred_arr.min())
    max_val = max(y_true_arr.max(), y_pred_arr.max())
    buffer = (max_val - min_val) * 0.05

    # Plot scatter plot and ideal fit line
    plt.scatter(y_true_arr, y_pred_arr, alpha=0.6, s=30, color='#228B22',
label=f'Dimension {i+1}')
    plt.plot([min_val - buffer, max_val + buffer],
             [min_val - buffer, max_val + buffer], 'r--', linewidth=2, label='Ideal Fit
Line')

    # Set axis limits
    plt.xlim(min_val - buffer, max_val + buffer)
    plt.ylim(min_val - buffer, max_val + buffer)

    # Chart annotations
    plt.xlabel('True Values', fontsize=11)
    plt.ylabel('Predicted Values', fontsize=11)
    plt.title(f'Output Dimension {i+1}\nR²: {metrics[f"Dimension {i+1}"]["R²"]:.4f} |
```

```
RMSE: {metrics[f"Dimension {i+1}"]["RMSE"]:.4f}',
                          fontsize=12, pad=10)
        plt.legend(fontsize=10)

plt.tight_layout()
plt.show()
```

1.4 The model code of RF

```
import pandas as pd
import numpy as np
import matplotlib.pyplot as plt
from sklearn.ensemble import RandomForestRegressor
from sklearn.model_selection import KFold, cross_validate, learning_curve
from sklearn.metrics import mean_squared_error, mean_absolute_error, r2_score

# Read data
file_path = r"C:\Users\gooddata.xlsx"
df = pd.read_excel(file_path, sheet_name="Sheet1")

# Split features and target variables
X = df.iloc[:, :22]
X.columns = X.columns.astype(str)
y = df.iloc[:, 23:27]

# Initialize Random Forest model
rf = RandomForestRegressor(n_estimators=100, random_state=42)

# Configure 10-fold cross-validation
kf = KFold(n_splits=10, shuffle=True, random_state=42)

# Define evaluation metrics
scoring = {
    'mse': 'neg_mean_squared_error',
    'rmse': 'neg_root_mean_squared_error',
    'mae': 'neg_mean_absolute_error',
    'r2': 'r2'
}

# Cross-validation evaluation for each dimension
results = {}
for i in range(y.shape[1]):
    print(f"\nEvaluation Metrics for Output Dimension {i+1}:")
    cv_results = cross_validate(rf, X, y.iloc[:, i], cv=kf, scoring=scoring)

    # Calculate average metrics (invert negative metrics)
    mse = -cv_results['test_mse'].mean()
    rmse = -cv_results['test_rmse'].mean()
    mae = -cv_results['test_mae'].mean()
    r2 = cv_results['test_r2'].mean()

    results[f'Dimension {i+1}'] = {'MSE': mse, 'RMSE': rmse, 'MAE': mae, 'R2': r2}

    print(f"MSE: {mse:.4f}")
    print(f"RMSE: {rmse:.4f}")
    print(f"MAE: {mae:.4f}")
    print(f"R²: {r2:.4f}")

```

```
# Train and evaluate on the full dataset
print("\nAverage Evaluation Metrics for All Dimensions:")
rf.fit(X, y)
y_pred = rf.predict(X)

for i in range(y.shape[1]):
    mse = mean_squared_error(y.iloc[:, i], y_pred[:, i])
    rmse = np.sqrt(mse)
    mae = mean_absolute_error(y.iloc[:, i], y_pred[:, i])
    r2 = r2_score(y.iloc[:, i], y_pred[:, i])

    print(f"\nDimension {i+1}:")
    print(f"MSE: {mse:.4f}")
    print(f"RMSE: {rmse:.4f}")
    print(f"MAE: {mae:.4f}")
    print(f"R²: {r2:.4f}")

# Visualization of predicted values vs true values
plt.figure(figsize=(15, 10))
plt.suptitle('Predicted Values vs True Values', y=1.02)

for i in range(y.shape[1]):
    plt.subplot(2, 2, i+1)
    plt.scatter(y.iloc[:, i], y_pred[:, i], alpha=0.6)
    plt.plot([y.iloc[:, i].min(), y.iloc[:, i].max()],
             [y.iloc[:, i].min(), y.iloc[:, i].max()],
             'r--', lw=2)
    plt.xlabel('True Values')
    plt.ylabel('Predicted Values')
    plt.title(f'Output Dimension {i+1}')

plt.tight_layout()
plt.show()

# Save prediction coordinate data
coords_df = pd.DataFrame({
    'True_Value_1': y.iloc[:, 0], 'Predicted_Value_1': y_pred[:, 0],
    'True_Value_2': y.iloc[:, 1], 'Predicted_Value_2': y_pred[:, 1],
    'True_Value_3': y.iloc[:, 2], 'Predicted_Value_3': y_pred[:, 2],
    'True_Value_4': y.iloc[:, 3], 'Predicted_Value_4': y_pred[:, 3]
})
# coords_df.to_excel(r"C:\Users\yanwq\Desktop\Prediction_Coordinate_Data.xlsx",
index=False)

print("\nExecution Completed!")

# Learning curve plotting function
plt.rcParams['font.family'] = 'Times New Roman'
def plot_learning_curve(estimator, X, y, cv=5, n_jobs=-1):
    train_sizes, train_scores, test_scores = learning_curve(
```

```
        estimator, X, y, cv=cv, n_jobs=n_jobs,
        scoring='neg_mean_squared_error',
        train_sizes=np.linspace(0.1, 1.0, 10)
    )

    train_scores_mean = -np.mean(train_scores, axis=1)
    test_scores_mean = -np.mean(test_scores, axis=1)

    plt.figure(figsize=(10, 6))
    plt.plot(train_sizes, train_scores_mean, 'o-', color="r", label="Training score")
    plt.plot(train_sizes, test_scores_mean, 'o-', color="g", label="Validation score")

    plt.xlabel("Training examples")
    plt.ylabel("Mean Squared Error")
    plt.title("Learning Curve")
    plt.legend(loc="best")
    plt.grid(False)
    plt.show()

# Plot learning curve for each dimension
for i in range(y.shape[1]):
    print(f"\nLearning Curve for Output Dimension {i+1}:")
    plot_learning_curve(rf, X, y.iloc[:, i])

# Collect cross-validation test set prediction results
cv_true = {f'Dimension {i+1}': [] for i in range(y.shape[1])}
cv_pred = {f'Dimension {i+1}': [] for i in range(y.shape[1])}

# Re-run cross-validation to collect data
kf = KFold(n_splits=10, shuffle=True, random_state=42)
for i in range(y.shape[1]):
    y_dim = y.iloc[:, i]
    for train_idx, test_idx in kf.split(X):
        X_train, X_test = X.iloc[train_idx], X.iloc[test_idx]
        y_train, y_test = y_dim.iloc[train_idx], y_dim.iloc[test_idx]

        rf.fit(X_train, y_train)
        y_test_pred = rf.predict(X_test)

        cv_true[f'Dimension {i+1}'].extend(y_test)
        cv_pred[f'Dimension {i+1}'].extend(y_test_pred)

# Visualization of cross-validation results
plt.figure(figsize=(15, 10))
plt.suptitle('Cross-Validation - Test Set: Predicted Values vs True Values (Generalization
Ability)', y=1.02)
for i in range(y.shape[1]):
    plt.subplot(2, 2, i+1)
    true_vals = cv_true[f'Dimension {i+1}']
    pred_vals = cv_pred[f'Dimension {i+1}']
```

```
        plt.scatter(true_vals, pred_vals, alpha=0.6)
        plt.plot([min(true_vals), max(true_vals)],
                 [min(true_vals), max(true_vals)],
                 'r--', lw=2)
        plt.xlabel('True Values')
        plt.ylabel('Predicted Values')
        plt.title(f'Output Dimension {i+1} (Cross-Validation)')
plt.tight_layout()
plt.show()
```
